# Strategies for managing the destruction of calcar femorale

**DOI:** 10.1186/s12891-021-04324-3

**Published:** 2021-05-19

**Authors:** Jin Mei, Lili Pang, Zhongchao Jiang

**Affiliations:** Hospital of Chengdu University of Traditional Chinese Medicine, Chengdu, 610072 Sichuan Province China

**Keywords:** Calcar femorale, Intertrochanteric fractures, Femoral neck fractures, Tumors

## Abstract

**Background:**

The calcar femorale was identified long ago. However, our current understanding of the calcar is insufficient, and its related concepts are sometimes confused. The calcar femoral is an important anatomical structure of the proximal femur, and its function can be overlooked. In trauma, tumors, or other diseases, the calcar femorale can be destroyed or changed pathologically. As a result, the mechanical structure of the proximal femur becomes destroyed, causing pathological fractures. How to address the destruction of the calcar femorale or the damage to the calcar femorale is discussed in this article.

**Main text:**

Destruction of the calcar femorale is accompanied by many conditions, including trauma, tumors, and other diseases. The types of hip fractures caused by trauma include femoral neck fractures and intertrochanteric fractures. Dynamic hip screws, proximal femoral nail anti-rotation, and multiple parallel cannulate pins can be used in different conditions. When metastatic and primary bone tumors involve the calcar femorale, endoprostheses are widely used. Other diseases, such as fibrous dysplasia and aneurysmal bone cyst are treated differently.

**Conclusions:**

The calcar femorale can redistribute stresses and the destruction of the calcar femorale can lead to an increase in posterior medial stress. Many factors need to be considered when deciding whether to reconstruct the calcar femorale. Effective treatment strategies for managing the destruction of calcar femorale will need first establishing the precise mechanism of the destruction of the calcar and then designing therapies towards these mechanisms. Further investigation to the calcar needs to be carried out.

## Background

The calcar femorale is an anatomical structure of the hip, its related concepts are often confused, and its clinical significance needs to be better understood. Wolff proposed the concepts called the “law of bone remodeling” and “the response of the bone”, where the anatomy of the bone is considered compatible with the biomechanics it bears [1, 2]. These concepts were explained further by Frost HM: modeling and remodeling of the bone are completed by osteoblastic drifts and osteoclastic drifts [3]. Thus, the calcar femorale plays an important role in the biomechanics of the proximal femur. The load transmitted from the femoral head to the upper femur does not follow a straight line considering the femoral neck-shaft angle and the anteversion angle. The force of the femoral neck under physiological loads is a combination of compressive stress, tensile stress, and shear force. The calcar femorale is sometimes destroyed in trauma or disease. Our understanding of the impact of the destruction of the calcar femorale is incomplete, and the optimal treatment strategy remains unclear.

## Main text

### The calcar femorale and its function

**Fig. 1:**
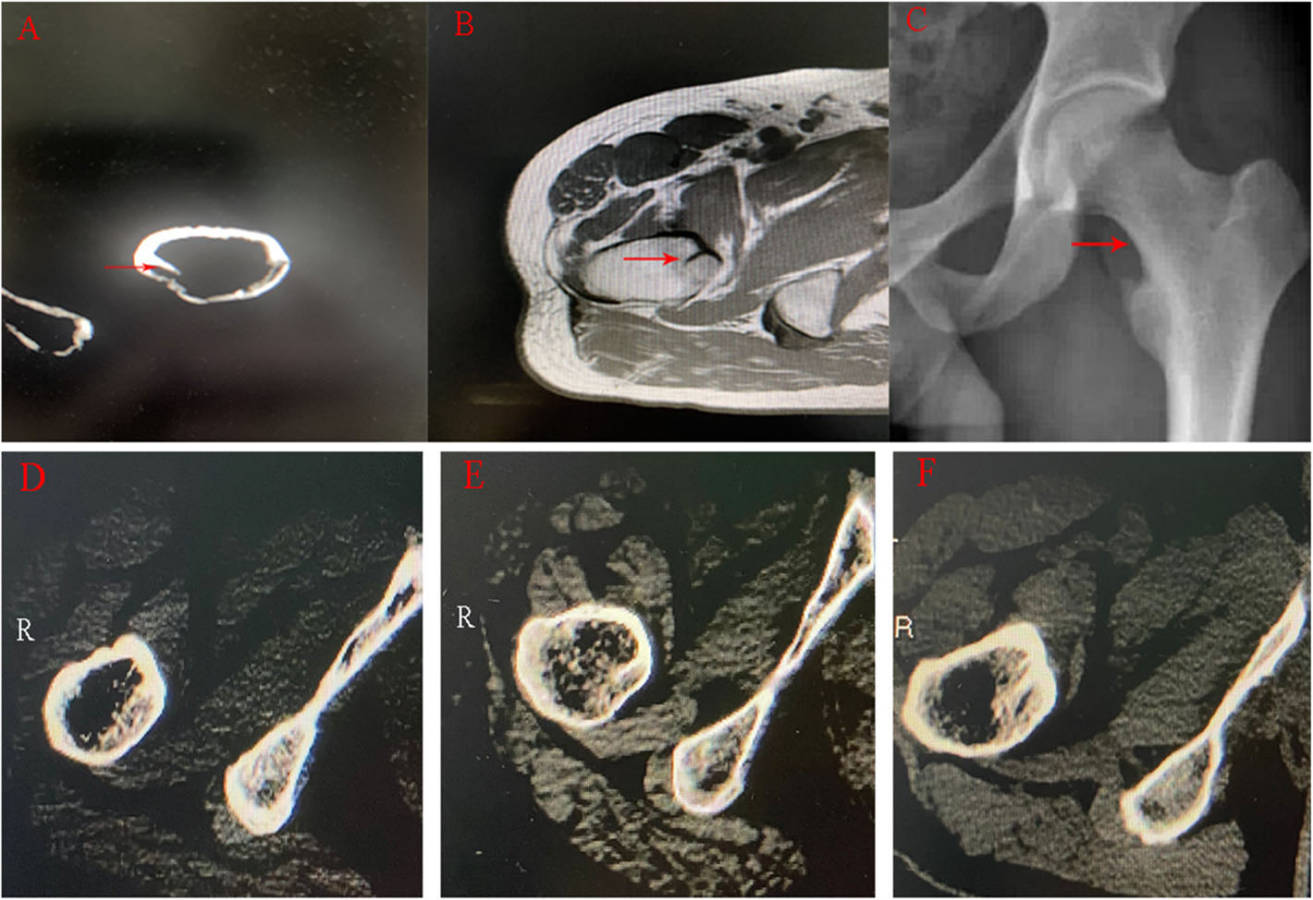
**a** The spur type of the calcar femorale can be seen in cross-sectional computed tomography images (red arrow) **b** The calcar femorale has a low signal in T1 weighted magnetic resonance images (red arrow) **c** The Adams arch (red arrow) is often confused with the calcar femorale. These three cross-sectional computed tomography images are from different individuals, the calcar femorale is classified into three types according to its length and thickness. **d** ridge-type: the calcar femorale is short and thick. **e** spur-type: the calcar femorale is longer than ridge-type and like a spur. **f** septum-type: the calcar femorale is thin and long

**Fig. 2:**
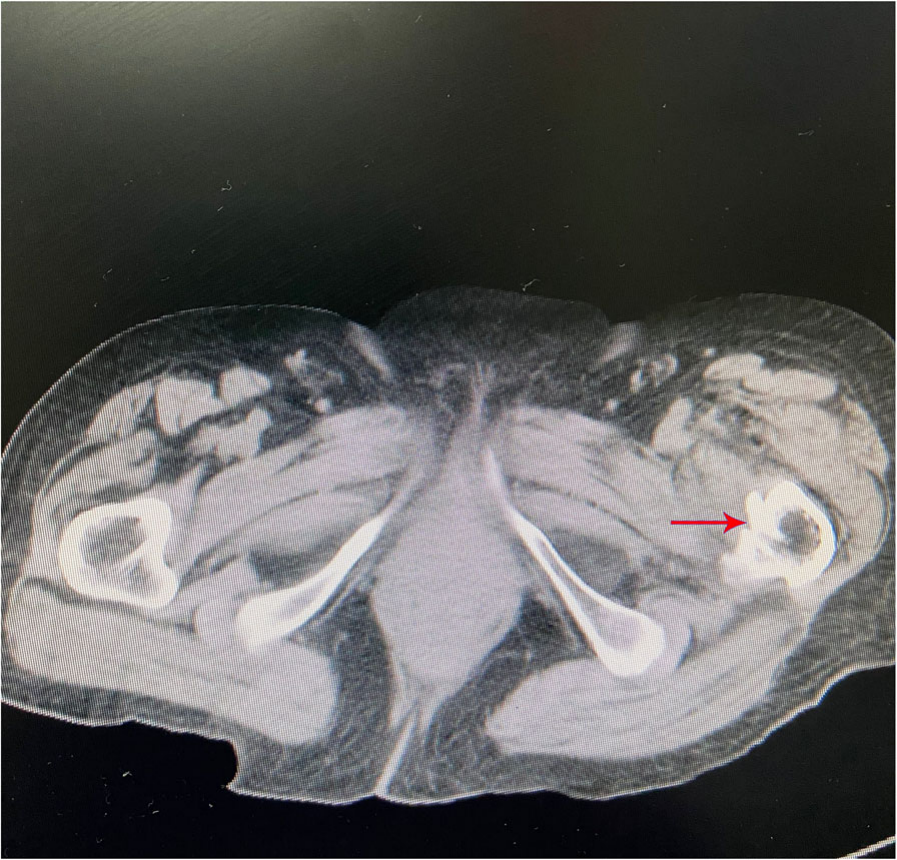
Destruction of the calcar femorale (red arrow) in cross-sectional computed tomography image of the right hip with an intertrochanteric fracture

The Discovery of the calcar femorale is a tortuous process [4]. The calcar femorale was first described in 1827. Charles B proposed that cancelli or minute lattice-work is an element of the interior structure of bone, and is related to the forces acting on the bone. In 1858, Humphry first described the calcar femorale as “cancelli radiating from the posterior wall of the neck”. The calcar femorale is complex (Fig. 1), the terms calcar, calcar area and calcar femorale are often used interchangeably. Calcar femorale and The Adams arch are often confused (Fig. 1c). The previous perception that the calcar femorale disappears when people become middle-aged proved to be wrong [5]. And lateral cortical thickness and the bone mass of the calcar femorale increases with weight and decrease with age in both men and women [6]. The density and stiffness of the calcar femorale are only slightly less than those of cortical bone from the mid-shaft of the femur in middle-aged and elderly individuals [7]. At the two-dimensional level, the calcar femorale can be divided into three types according to its size on CT cross-sectional images: the ridge type, spur type, and septum type (Fig. 2). Le Corroller et al. describe the calcar as existing in three different shapes of different sizes, the mean length being 3 mm, height 9.94 mm and thickness 2.71 mm [8]. Hammer A gave a more detailed description: the calcar femorale lies directly under the upper part of the lesser trochanter. Medially it is attached to the lower part of the vertical trabecular column. Superiorly it has an attachment with the horizontal trabeculae column. Laterally it attaches to the protruding buttress [9].

Regarding the function of the calcar femorale, the calcar femorale and the three types of bone trabeculae consist of compression, oblique and tension trabeculae, forming the loading system-truss system. The three types of bone trabeculae increase the rigidity of the passing area by 160 to 400%. Zhang Y found that the compression forces were mainly concentrated on the medial surface of the femur by a finite element analysis [10]. The anterior wall of the femur at the calcar’s level is strong and thick but the posterior wall is otherwise. Bigelow even proposed that the calcar femorale is the true posterior wall of the upper femur [11]. Although people have different opinions on aspects of the calcar femorale (Table 1), its functional support and mechanical transmission properties at the proximal femur cannot be ignored. Farkas et al. suggested that the calcar femorale is an internal weight-bearing system of the proximal femur [12]. Li B proposed that the calcar femorale is a supporting structure to the femoral neck and can transfer stress from the trabecular bone of the femoral head and neck to the femoral shaft [13]. The posterointernal parts of the proximal femur bear more strain than do the anterolateral and calcar femorale parts, which play a significant role in redistributing stress along the proximal femur [14]. The stress is redistributed by decreasing the load in the posterointernal aspects and increasing the load in the anterolateral aspects. Therefore, the calcar femorale is a ridge of dense bone along the posteromedial endosteal surface of the proximal femoral shaft near the lesser trochanter. It is vertical in orientation, and it projects laterally toward the greater trochanter. The calcar femorale provides mechanical support and plays an important role in load distribution within the proximal femur [15].

**Table 1:** Different viewpoints on the function of the calcar femorale

Authors	Years	The function of calcar femoral
Farkas et al. [12]	1948	An internal weight bearing system of the femoral neck.
Li and Aspden [13]	1998	A supporting structure to the femoral neck and can transfer stress from the trabecular bone of the femoral head and neck to the femoral shaft
Zhang et al. [14]	2009	Bears compression load and redistributes stress or load from the femoral head to the proximal femur

### Fractures

Hip fracture patients have a high risk of death compared to the general population [16]. For proximal femoral fractures with an intact calcar femorale, it is better to fix the internal fixation device at a position close to the calcar femorale. This method not only strengthens the calcar femorale but also preserves the distribution of compression forces and load transmission of the proximal femur. The destruction of the calcar femorale has a large impact on the mechanical structure of the entire femur, thereby affecting the optimal procedures. The calcar femorale is a frequently mentioned concept in the treatment of intertrochanteric and femoral neck fractures. We often hear phrases such as “nailing against the calcar femorale”, “ carefully protect the calcar femorale” and “the calcar is destroyed”. specific analysis is described below.

### Intertrochanteric fractures

Intertrochanteric fractures often result in a shattered calcar near the junction between the femoral shaft and the lesser trochanter (Fig. 2). Xiong et al. reported in a morphologic study that 87% of the lesser trochanter fragment contains the calcar femorale [17]. Naimark A proposed that the stability *of intertrochanteric* fractures depends on the obliquity *of* the fracture line and the critical calcar area [18]. Seker A also found a high level of stress at the calcar femorale after proximal femoral nail fixation of trochanteric fractures when assessing the effects of early weight bearing [19].

Evans classification system is commonly used for intertrochanteric fractures. Harris WH et al. implemented mechanical tests in intertrochanteric fractures with fracture masses of different sizes in the posterior medial region. He found that fixation of the posteromedial fragment, especially the calcar femorale, is important to the structural stability of hips [20]. DHSs (Dynamic Hip Screws) may be a better choice for stable fractures of Evans types I and II. When DHSs have applied in Evans type III fractures, screw bending often occurs because compressive stresses cannot be transmitted through the calcar femorale. The destruction of the calcar femorale affects the crucial weight-bearing area of the proximal femur and leads to the unsuccessful treatment of unstable intertrochanteric fractures [21]. Compared to DHSs, proximal femoral nail anti-rotation (PFNA) is a satisfactory fixation method for unstable intertrochanteric fractures [22]. However, a poor calcar femorale restoration is also one of the risk factors for the failure of unstable intertrochanteric fractures after PFNA [18]. The calcar fractures also increase the risk of revision after cementless total hip arthroplasty [23]. Thakkar CJ found that 94% of patients who underwent hemiarthroplasty with calcar femorale grafting achieved a satisfactory outcome with few complications [24]. An integral calcar femorale is critical to maintaining the stability of the internal fixators. Therefore, satisfactory *calcar* reduction can lead to a satisfactory outcome and decrease the risk of complications [25]. However, Zha GC holds different views: for elderly patients with unstable intertrochanteric fractures, bipolar hemiarthroplasty using cementless distal fixation modular prostheses achieves satisfactory mid-term radiographic and clinical outcomes without reconstruction of the calcar femorale [26].

### Femoral neck fractures

Femoral neck fractures with the destruction of the calcar femorale are rarely seen in clinical practice and are easily overlooked. The optimal surgical treatment for femoral neck fractures remains controversial. The degree of displacement and comminution of the *calcar femorale*, varus angulation and size of the femoral head can affect the effects of treatment for femoral *neck* fractures. DHSs and multiple parallel cannulate screws are the most commonly used internal fixation techniques. Multiple parallel cannulate screws can prevent secondary displacement and reduce the risk of femoral head necrosis. However, how to place the screw at the best position in the limited intramedullary space still needs to be discussed. Placement of the cannulate screws close to the posterior cortex and calcar femorale can decrease the risk of reoperation. From Lindequist’s viewpoint, the rationale for placing the fixation device close to the calcar in femoral neck fractures is that the fracture will subside until the cannulated screws abut the intact cortex of the calcar femorale [27, 28]. In a study of proximal femur morphology, Nakanishi Y also found that the anterior cannulate screw position affected the calcar [29]. Filipov O proposed that biplane double-supported screw fixation (BDSF) with two cannulate screws buttressed on the calcar can provide additional cortical support [30]. In the treatment of high-shear Pauwels III femoral neck fractures with cannulated screws, Tianye L compared four different internal fixations and found that the “F” cannulate screw technique may be a good method. *It can* eliminate shear and torsional stresses and exert compressive stresses on *the fracture* end, but more clinical trials are needed to confirm this finding [31]. In a study comparing 3 different cannulate screw configurations for unstable femoral *neck fractures*, a transverse screw in the *calcar* was shown to provide a more stable structure [32]. Satisfactory reduction of the calcar femorale can minimize complications and improve functional outcomes [33]. For patients with posteromedial calcar fragments, the proximal end of the DHS is fixed with a cannulated screw also can obtain anti-rotation stability and good support [34].

### Intraoperative fractures

The operation can also lead to the destruction of the calcar femorale. The calcar crack was first described by Mont et as the most common intraoperative fracture [35]. Intraoperative fractures of the calcar femorale are often occurred in cementless total hip arthroplasty (THA) [36]. Its incidence varies from 0.1 to 11% [37, 38]. Intraoperative fractures with uncemented components often occur in patients with dysplastic femurs and women [39, 40]. The proximal femoral shape also affects the risk of intraoperative fractures of the calcar femorale. Patients who have smaller and narrower femurs are more susceptible to an intraoperative fracture of the *calcar* [41]. For deviant-shaped proximal femurs, cementless stems should be installed with care to avoid artificial damage to the calcar femorale. Cerclage wires or cables may be a good method for stabilizing calcar crack [42]. Because it can resist the stresses of axial and rotational on the stem [43].

### Tumor

**Fig. 3:**
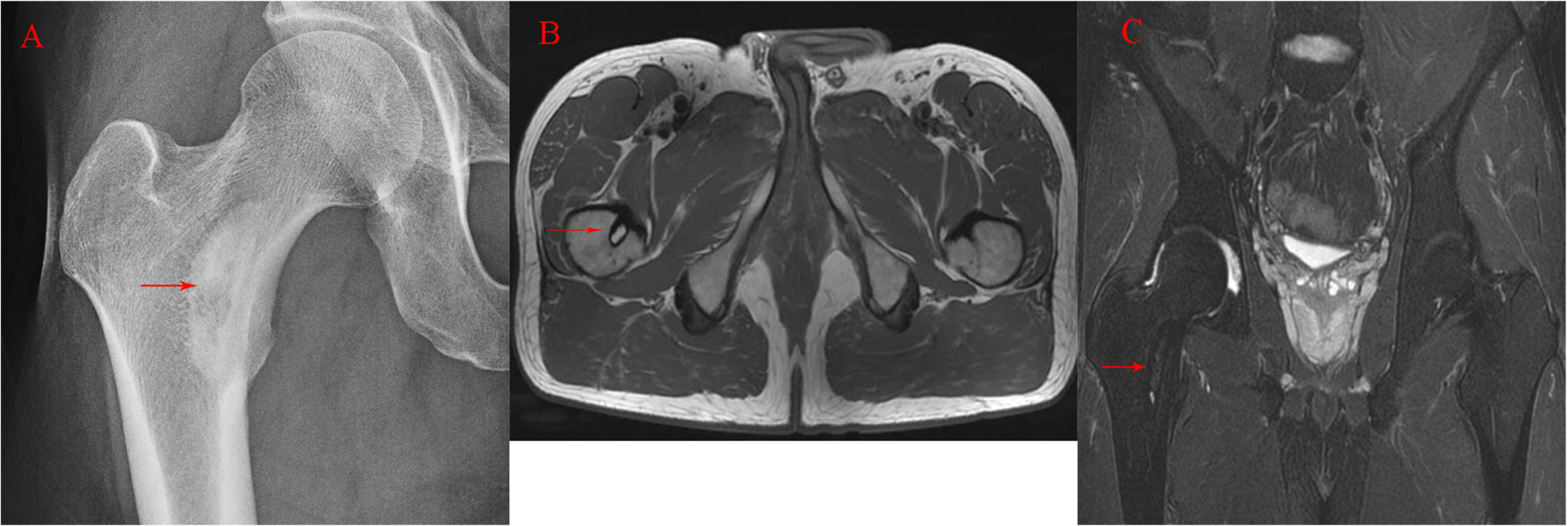
**a** An enchondroma is located in the calcar femorale in the coronal-view plain radiography (red arrow) **b c** It is also can be seen in a transverse plane of T1 weighted magnetic resonance image and the coronal plane of T2 weighted magnetic resonance image respectively

The proximal femur is a common site for primary bone tumors and influences the mechanical structure of the proximal femur (Fig. 3). Patients are typically diagnosed with a tumor of the proximal femur after they had a pathological fracture [44]. Patients diagnosed with proximal femur tumors have different demographic characteristics, and their oncologic survival rates are different. With an extended survival time, many survivors expect to have an active lifestyle and good quality of life. Therefore, the improvement on function and implant longevity is more important after the proximal femur is resected [45, 46]. However, there aren’t standardized guidelines or clinical study results that can guide the choice of appropriate operative procedures. Osteosynthetic fixation and prosthetic replacement are common operative procedures [47, 48], but which procedure is better is still debated. Life expectancy and the performance status before fracture are the main factors affecting the operative procedures [49]. and another important factor, the degree of bone destruction, was discussed in one study [50]. prosthesis applies to the following bone destruction factors: 1. Involvement of the head, neck, calcar femorale, and intertrochanteric region 2. Transverse destruction > 50% 3.Tumor extension to soft-tissue. Cho HS reviewed 7 patients with tumors involving the proximal femur: conventional total hip prostheses with *calcar* preservation yielded satisfactory outcomes [51].

### Tumor-like lesions

Fibrous dysplasia is a nonhereditary benign disease in which normal bone tissue is replaced by abnormally hyperplastic immature reticular bone and fibrous tissue. It can be divided into three clinical types: monostotic, polyostotic and McCune-Albright syndrome [52]. When the lesion progresses to the proximal part of the femur, the mechanical forces lead to progressive varus and bowing deformities or pathological fractures in most patients [53]. Guille JT declared that the calcar was not involved in cases with a monostotic lesion in his study group and these patients had less severe deformities. However, for patients who have polyostotic lesions, and the calcar is often involved, medial displacement osteotomy is an effective treatment [54].

Aneurysmal bone cysts (ABCs) are benign single bone tumors characterized by a uniform foam-like translucent area within the tumor. Weber MG founded that one patient had a pathologic fracture when the lesion area occurred at intertrochanteric and calcar femorale [55]. The accepted treatment is curettage and bone grafting when pathologic fractures occur [56]. However, internal fixation is mandatory when the fracture reaches the critical weight-bearing area. Curettage with bone grafting and cephalomedullary nail fixation may be an effective treatment for patients with a pathological fracture secondary to ABC.

## Discussion

**Fig. 4:**
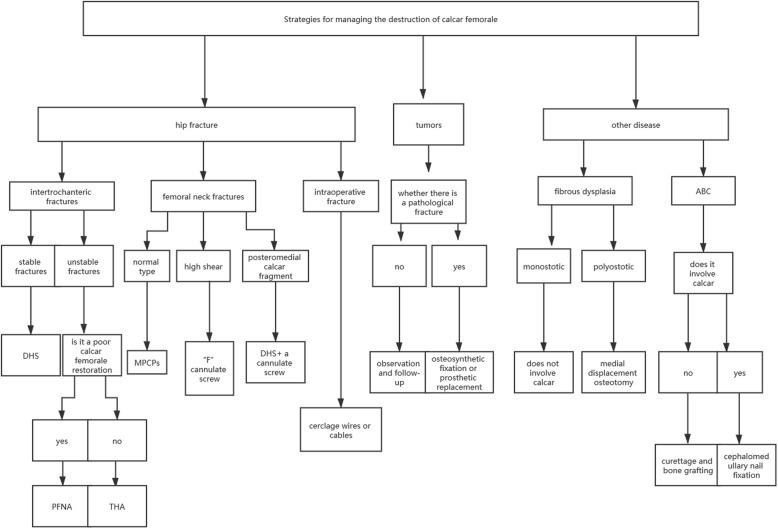
Strategies for managing the destruction of the calcar femorale. DHS: dynamic hip screw; PFNA: proximal femoral nail anti-rotation; THA: total hip arthroplasty; MPCPs: multiple parallel cannulate pins; ABC: Aneurysmal bone cyst

The calcar femorale plays a core role in dealing with the complex forces that occur at the junction of the femoral shaft and neck. It is an important part of the internal weight-bearing structure of the upper femur. It also can redistribute the stresses of the proximal femur, reduce the posterior and medial cortical loads and increase the anterior and lateral cortical loads, and make the process of transmitting stress from the femoral head to the femoral shaft more reasonable. Reduction of the calcar femorale can effectively improve the mechanical transmission of the proximal femur. Strategies for managing the destruction of the calcar femorale are shown in the flow chart below (Fig. 4). In hip fractures, the type of fracture should be considered first, and different procedures can be selected according to whether the calcar is involved. As for tumors, life expectancy is the main factor in deciding whether to operate and the degree of bone destruction should also be evaluated. Different procedures can be selected in other diseases involving the calcar.

Clinical studies have revealed that the incidence of intertrochanteric fractures gradually increases with age [57, 58]. Some scholars believe that this finding is related to the calcar femorale. The density and stiffness of the calcar femorale are only slightly less than those of cortical bone from the mid-shaft of the femur in middle-aged and elderly individuals. The femoral trochanter is the place with the lowest bone mineral density in the hip and the most sensitive part of bone loss. Low bone mass and fragile bone structures are the main cause of hip fractures in this region [59]. Some researchers believe that for every increase in bone density by one standard deviation, the risk of fracture increases by 2 to 3 times [60]. So bone mass can also be used as a predictor for hip fractures [61]. There are no significant differences in the density or rigidity of the calcar femorale between osteoporosis patients and normal people. Therefore, it is osteoporosis rather than changes in the calcar femorale that increases the risk of intertrochanteric fractures.

For stable femoral neck fractures, multiple cannulate screws are commonly used for internal fixation [62]. However, putting the screw in the best position is still needs discussion in the limited intramedullary space. Lindequist put forward that the cannulate screws close to the calcar can accelerate the healing of the fracture, and Filipov O also proposed that two cannulate screws buttressed on the calcar can provide additional cortical support. However, for unstable femoral neck fractures (Pauwels III), a study proposed that a transverse screw in the *calcar* can provide a more stable structure after comparing 3 different cannulate screw configurations. Tianye L compared four different internal fixations and found that the “F” cannulate screw technique may be a good method. When using DHSs to treat unstable intertrochanteric fractures, putting the screw posterior and inferior to the femoral head may help support the posteromedial cortex and calcar femorale and decrease the risk of cut-out [63]. It is recommended that the proximal end of the DHS is fixed with a cannulated screw. Therefore, the relationship between internal fixation and the calcar femorale is strong.

The primary complication of THA remains aseptic loosening [64]. Periprosthetic bone loss and bone resorption are crucial factors that lead to aseptic loosening [65]. The calcar femorale prevents aseptic loosening and subsidence. Some scholars believe that the femoral neck should be kept 1.0 ~ 1.5 cm above the trochanter, which can prevent the destruction of the calcar femorale, thereby preventing the subsidence of prosthesis, and reducing the incidence of hip varus and length discrepancies between the lower limbs. Many scholars have found that preserving the calcar femorale or rebuilding the calcar during THA can establish a more stable mechanical structure. However, due to the periprosthetic bone loss caused by the stress shielding effect, the medial calcar is the most affected region. Femoral stem design can influence the degree of stress shielding. Advancements in stem material, geometry and design are crucial for restoring a physiologic load transfer through the calcar femorale. Calcar-guided short stems can better adapt the anatomical curvature of the calcar femorale in modern THA than can conventional straight-stem designs. However, the long-term clinical effects need to be studied. In some cases of femoral neck shortening, short stems should also be used, and the calcar femoral should not be removed heavily so that the tension of the gluteus medius muscle can be maintained and subsidence can be prevented. Contacting the collar of a femoral prosthesis with the calcar femorale can increase the vertical stress within the region of the calcar femorale. Calcar collars can improve stability by exerting compressive loads on the calcar and are often used on cementless stems to prevent the subsidence of prostheses [66]. However, some scholars hold different views. Wroblewski BM proposed that clearing the calcar can provide an adequate cement mantle and decrease the risk of revisions due to aseptic loosening [67]. Zhang GC used bipolar hemiarthroplasty to treat intertrochanteric fractures without reconstruction of the calcar femorale and achieved satisfactory clinical and radiological outcomes. However, preventing subsidence of the stem and maintaining the stability of the implant is a challenge without the calcar femorale.

The calcar femorale has also been used as an important predictive parameter. A cut-out is a common complication after DHSs in intertrochanteric fractures [68]. A tip-apex distance (TAD) less than 25 mm is recommended to decrease the risk of screw cut-out [69]. Later, a new concept of calcar-referenced tip-apex distance (CalTAD) was proposed [70] and is known as a significant cut-out predictor [71]. However, which predictor is better has been debated. CalTAD can also be utilized for helical blade placement. In patients with developmental dysplasia of the hip, the preoperative calcar femorale angles at the low femoral neck can be effective parameters to predict postoperative stem anteversion [72].

There is no unified conclusion about the optimal method of calcar femorale reconstruction. Accordingly, there are varied reconstruction materials, including bone cement, metal mesh, steel plates, steel wire binding and vascularized femoral flaps. Currently, the most commonly used methods include bone cement reconstruction and femoral calcar fracture reduction. A study involving three-dimensional finite element analysis showed that the distribution of stresses was more uniform and that the biomechanics were more stable with bone cement reconstruction. But which treatment method is better needs to be studied extensively.

## Conclusions

The calcar femorale can redistribute stresses and the destruction of the calcar femorale can lead to an increase in posterior medial stress. Many factors need to be considered when deciding whether to reconstruct the calcar femorale. Effective treatment strategies for managing the destruction of calcar femorale will need first establishing the precise mechanism of the destruction of the calcar and then designing therapies towards these mechanisms. Further investigation to the calcar needs to be carried out.

## Data Availability

Data sharing is not applicable to this article as no datasets were generated or analysed during the current study.

## Abbreviations

DHSs: dynamic hip screws
PFNA: proximal femoral nail anti-rotation
BDSF: biplane double-supported screw fixation
THA: total hip arthroplasty
ABCs: Aneurysmal bone cysts
TAD: tip-apex distance
CalTAD: calcar-referenced tip-apex distance
